# Biomechanical Alterations in the Unweight Phase of the Single-Leg Countermovement Jump After ACL Reconstruction

**DOI:** 10.3390/jfmk10030296

**Published:** 2025-07-30

**Authors:** Roberto Ricupito, Marco Bravi, Fabio Santacaterina, Giandomenico Campardo, Riccardo Guarise, Rosalba Castellucci, Ismail Bouzekraoui Alaoui, Florian Forelli

**Affiliations:** 1 Restart Physiotherapy, Via Felice Grossi Gondi 35, 00162 Rome, Italy; robertoricupito90@gmail.com; 2 Fondazione Policlinico Universitario Campus Bio-Medico, Via Alvaro del Portillo, 200, 00128 Rome, Italy; m.bravi@policlinicocampus.it (M.B.); f.santacaterina@policlinicocampus.it (F.S.); 3 Campardo Private Clinic, Via S. Marco 10, 33170 Pordenone, Italy; giandomenico.campardo@gmail.com; 4 Physiotherapy Unit, CF Research Centre, University Hospital of Verona o Azienda Ospedaliera Unviersitaria Integrata di Verona, 37126 Verona, Italy; riccardo.guarise@aovr.veneto.it; 5 Castellucci Private Clinic, Via Maiella, 75023 Montalbano Jonico, Italy; castellucci.ross@gmail.com; 6 Mohammed VI University of Sciences and Health-UM6SS, Casablanca 20270, Morocco; alaoui.ismail.bz@gmail.com; 7 Haute-Ecole Arc Santé, HES-SO University of Applied Sciences and Arts Western Switzerland, 2800 Delémont, Switzerland; 8 Orthopaedic Surgery Department, Clinic of Domont, Ramsay Healthcare, OrthoLab, 95330 Domont, France; 9 Société Française des Masseurs—Kinésithérapeutes du Sport Lab, 93380 Pierrefite sur Seine, France

**Keywords:** ACL reconstruction, countermovement jump, biomechanics, neuromuscular control, rehabilitation, force plate

## Abstract

**Background:** Anterior cruciate ligament reconstruction (ACLr) often leads to asymmetries between limbs, with variable return-to-performance rates in athletes. The single-leg countermovement jump (SLCMJ) is commonly used to assess postoperative knee function. However, limited research has explored deficits specifically during the unweighting phase of the jump. **Methods:** This study assessed 53 recreational athletes (11 females, 42 males) between 6 and 9 months post-ACLr using a dual force plate system (1000 Hz). Each participant performed three maximal-effort SLCMJs per limb. Outcome measures included jump height, negative peak velocity, minimum force, and center of mass (COM) displacement. Paired *t*-tests and Wilcoxon tests were used to compare the ACLr limb with the contralateral limb. **Results:** Compared to the healthy limb, the ACLr limb showed significantly lower negative peak velocity (−0.80 ± 0.40 m/s vs. −0.94 ± 0.40 m/s, *p* < 0.001), higher minimum force (36.75 ± 17.88 kg vs. 32.05 ± 17.25 kg, *p* < 0.001), and reduced COM displacement (−17.62 ± 6.25 cm vs. −19.73 ± 5.34 cm, *p* = 0.014). Eccentric phase duration did not differ significantly. **Conclusions:** Athletes post-ACLr demonstrate altered neuromuscular control during the early SLCMJ phase. These findings highlight the importance of rehabilitation strategies targeting eccentric strength and symmetry restoration.

## 1. Introduction

Anterior cruciate ligament (ACL) injury is a significant concern across all sports. The rate of return to performance after ACL reconstruction ranges from 55% to 81% [1,2], while the risk of re-injury varies between 1.5% and 37.5% [3]. ACL injuries are commonly classified as deceleration injuries, typically occurring during landing or change-of-direction movements, and are often associated with external perturbations like cognitive (like a head fake) or physical (like an upper body push from an opposite player while landing) [4]. Deceleration ability is closely related to the eccentric function of the neuromusculoskeletal system, which is responsible for absorbing and dissipating kinetic energy from the body, and in particular, during a vertical jump, the knee is one of the most important joints in the energy storage and release [5]. Therefore, it is important to characterize the unweighting and braking function of both limbs (reconstructed and non-reconstructed) during the return-to-sport process in order to assess potential deficits in the ACL-reconstructed limb (ACLr). Such deficits can lead to increased loading of the non-reconstructed limb and may contribute to contralateral ACL injuries.

An inability to move rapidly and achieve sufficient depth during movement may be attributed to deficits in eccentric strength, persistent quadriceps inhibition, or excessive compliance of connective tissues [6]. These factors can impair the patient’s ability to fully relax agonist muscles and flex the hip, knee, and ankle, ultimately reducing the amount of force absorbed during the braking phase and diminishing the stretch-shortening cycle [7,8].

**Figure 1:**
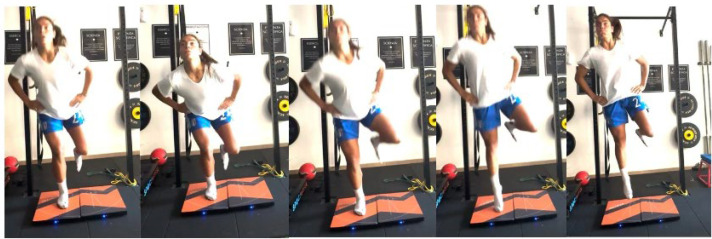
Execution of the single-leg countermovement jump (SLCMJ). The participant begins in an upright position, performs a rapid downward movement (eccentric phase), and then immediately jumps vertically off a single leg (concentric phase), aiming to achieve maximal height. Hands were placed on the hips to minimize upper body contribution.

In this context vertical jump tests could be used since they can provide information about deceleration abilities [9,10,11]. As a matter of fact, vertical jump tests are among the most widely used assessments in the literature to evaluate knee readiness after ACL reconstruction (ACLr), as they optimally emphasize knee function over hip function [12,13]. One of the most commonly utilized vertical jumps is the single-leg countermovement jump (SLCMJ) (Figure 1), which is characterized by distinct phases of force development. The initial phase involves unweighting, followed by a downward phase to a semi-squat position, an upward phase with forceful triple extension of the ankle, knee, and hip, a flight phase, and finally, a landing phase [14,15].

The SLCMJ can provide information about the neuromuscular strategy during a slow stretch shortening cycle action (i.e., coupled eccentric–concentric force generation) and has shown correlations with deceleration, change-of-direction, and maximal strength abilities [9,10,11]. Various SLCMJ-related metrics derive from kinetic and kinematic variables, which explain primary outcome measures such as jump height or take-off velocity. The gold standard for assessing these metrics is the force plate, which objectively measures numerous parameters across different tasks [16]. For these reasons, the SLCMJ is considered an optimal tool for assessing injury recovery status, neuromuscular fatigue, or the performance profile of an athlete. Additionally, it is easy to administer and demonstrates good to excellent reliability [17,18,19].

It is well established that athletes show persistent deficits at the time of return to sport, both in strength and vertical jump performance, including reduced vertical ground reaction force, jump height, concentric impulse and reactive strength [20,21,22,23,24,25]. However, to date, no studies have specifically evaluated differences between the ACLr and contralateral (healthy) limb in terms of negative velocity, peak minimum force, and center of mass displacement between 6 and 9 months after ACLr. This gap is critical, as these factors may represent key elements to consider during different stages of rehabilitation. Therefore, the aim of this observational study is to assess the differences between the ACLr limb and the contralateral limb in negative velocity, peak minimum force, and center of mass displacement between 6 and 9 months after ACLr. The hypothesis is that persistent deficits will be present in the ACLr limb.

## 2. Materials and Methods

### 2.1. Ethical Consideration and Study Design

This multicenter retrospective study was conducted between December 2023 and January 2024 across two physiotherapy clinics in Rome (Italy). The study received the Ethical Committee of Ramsay Healthcare approval (IRB00010835) and was held in accordance with the Declaration of Helsinki.

Existing literature lacks data on the primary outcomes, including variability, group differences, or other key parameters needed for sample size calculation using standard methods. A Monte Carlo simulation was conducted based on hypothetical population mean and standard deviation values. Since the existing literature does not provide established normative data for the SLCMJ test parameters in ACLr, the simulation modeled a hypothetical distribution representative of the general population. Simulation parameters were as follows: (1) hypothetical population mean: 15.274; (2) standard deviation: 4.034; (3) clinically relevant difference to detect: 5.0; (4) number of simulations: 10,000 iterations; (5) significance level: 0.05. In each simulation, random samples of size n = 53 were repeatedly drawn from the simulated distribution. The mean and SD for each sample were calculated to assess variability and sampling error. The results demonstrated that the collected sample exhibited comparable variability to that expected in simulated samples, suggesting that the sample size provides a sufficiently robust and representative estimate of the parameters of interest.

### 2.2. Inclusion and Exclusion Criteria

The following inclusion criteria were used: adult patients (age > 18 years); between 6 and 9 months after primary ACLr, grafts obtained from patellar tendon, hamstring tendon, or quadriceps tendon, with or without meniscal repair or meniscectomy. Furthermore, we include patients with a Tegner Activity Scale from level 5 to 10, including both competitive and non-competitive athletes.

Exclusion criteria were patients with allograft reconstructions, revision procedures, contralateral ACLr, or those who underwent additional knee surgeries (cartilage, multiligament, etc.).

### 2.3. Assessments and Instruments

**Figure 2:**
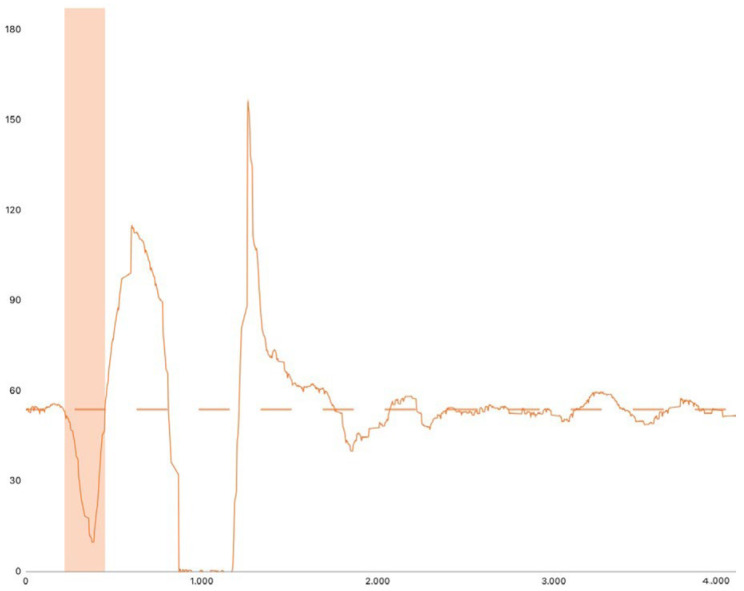
Force–time curve phase. The orange block represents the unweighting phase of the SLCMJ.

The unweighting phase (Figure 2) of the SLCMJ begins when the athlete initiates a countermovement by relaxing the agonist muscles, leading to hip and knee flexion along with dorsiflexion. This phase starts when vertical force decreases below a defined threshold. The onset of movement is described as the point where vertical force drops by 5 times the standard deviation (SD) of body weight (BW) measured during the weighing phase, emphasizing the importance of minimizing BW variability during this phase [26]. Owen et al. [26] also recommended considering a point 30 ms earlier than the detected onset, as movement likely begins before the force drop is detected. A small error (5–10 ms) in identifying the onset has minimal impact (<0.1%) on center of mass (COM) velocity and displacement calculations due to the gradual force change at this stage. However, inaccuracies can significantly affect time-dependent variables such as time to take-off and rate of force development. To ensure comparability, a consistent threshold should be applied across trials and athletes. The unweight phase ends when the patient reaches the peak negative velocity and continues with the deceleration phase till the velocity reaches 0 m/s [26].

Participants completed a standardized warm-up that included 10 min of light cycling on an ergometer and 2 sets of 10 bilateral squats, lunges, and push-ups; 2 submaximal-effort SLCMJs, with each jump separated by 3 s of quiet standing; and after the familiarization, participants performed 3 maximal-effort SLCMJs starting with the healthy leg. To encourage maximum vertical jump height (VJH), participants received the instruction “go down and up as fast as you can and jump as high as you can”. Jumps were performed with hands placed on the hips and the free leg at 45° of hip-knee flexion to minimize leg swing; participants were free to select their preferred countermovement depth [24,27]. After every repetition, the patient rests in place for 30 s before starting the next repetition. To avoid leg swing, they were asked to keep the non-jumping leg at 45° of hip and knee flexion. A summary of the test procedures is outlined in Table 1. All the patients recorded two SLCMJs, and the best jump for the healthy and ACLr legs (highest jump) was taken for the analysis [11,20]. All tests were collected from two sports physical therapists with at least 3 years of experience in the field of force plate assessment and ACLr rehabilitation.

The vertical ground reaction force (Fz) for both the healthy and ACLr legs was simultaneously collected using a dual force plate system (Deltas V3, Kinvent^®^, Montpellier, France) at a sampling frequency of 1000 Hz. Prior to each testing session, plates were tared with no load to ensure baseline correction. The system undergoes internal auto-calibration upon startup. This system has demonstrated good concurrent validity and reliability for vertical jump assessments [28].

In addition to the data from SLCMJ tests, information was also collected on activity levels using the Tegner Activity Score (TAS), as well as sample characteristics such as age, gender, weight, height, type of graft, time since ACLr, and type of sport practiced.

### 2.4. SLCMJ Metrics

For analyzing vertical jump performance, the flight time method measures airborne duration to derive height, while the impulse–momentum approach, often considered the gold standard, uses force and contact time to calculate take-off velocity and subsequently jump height [6]. VJH was calculated with the impulse–momentum relationship with the following formula:

$$\mathrm{VJH}=\frac{\mathrm{Take}\;\mathrm{Off}\;{\mathrm{Velocity}}^{2}}{2\times 9.81m/s^{2}}.$$

Negative peak velocity is derived by integrating the acceleration (a) data over time, where *t*_0_ and *t*_1_ represent the start and end of the unweighting phase, focusing on the period when the body decelerates toward the lowest position before the upward phase of the jump [13].

$$v_{\mathrm{peak-negative}}=\int_{t_{0}}^{t_{1}}a\left(t\right)dt.$$

Minimum force is identified as the lowest vertical ground reaction force (*F_z_*) exerted on the platform during the unweighting phase, which corresponds to the reduction in force as the jumper moves downward, where *F_z_*(*t*) is the vertical ground reaction force at time *t* within the unweighting phase [13].

$$F_{\min}=\min(F_{z}(t))\mathrm{for}\;t\in [t_{0},t_{1}].$$

The eccentric phase duration was defined as the time interval starting from the moment when the vertical ground reaction force returned to the participant’s body weight, indicating the end of the unweighting phase, until the point at which the center of mass velocity reached zero.

Finally, COM displacement (Δ*y*COM) is calculated by integrating velocity *v*(*t*) data to track the downward movement of the center of mass until the lowest point is reached, where *v*(*t*) is the velocity at time t, derived from the previous integration of acceleration, with the limits t0 and t1 marking the duration of the unweighting phase [13].

$$∆y\mathrm{COM}=\int_{t_{0}}^{t_{1}}v\left(t\right)dt$$

### 2.5. Statistical Analysis

A descriptive analysis was performed examining all anthropometric (sex, age, weight, etc.) and clinical variables (type of graft surgery, days from surgery, sport activity and TAS of the recruited patients). This analysis included data such as mean, SD, median, and interquartile range, depending on the nature of the data (continuous or categorical variables). The data obtained from the assessments were collected in a database created using Excel (Microsoft Office 2016) and subsequently prepared for statistical analysis using STATA software, Version 18 (StataCorp, Texas Ltd., College Station, TX, USA). After testing the normal distribution of the data using the Shapiro–Wilk test, appropriate statistical tests were applied to determine the presence of significant differences in jump height, peak negative velocity, minimum force, COM displacement and eccentric phase duration of SLCMJ between ACLr and healthy legs. The paired Student’s *t*-test was used for comparing the means of variables with a normal distribution, while the non-parametric Wilcoxon test was employed for variables that did not meet the assumption of normality. The variables “negative velocity peak” and “eccentric phase duration” of both the injured and healthy legs do not follow a normal distribution (*p* < 0.001). Analysis of variance (ANOVA) was performed to assess significant differences in the variables under investigation. In cases where variables were not normally distributed (e.g., mean distance), a rank-based comparison was carried out using the Kruskal–Wallis test. Given the exploratory nature of the study, analyses were performed without correction for multiple comparisons. A significance level of *p* < 0.05 was considered.

## 3. Results

A total of 52 patients (11 females and 41 males) who had undergone ACLr were included in the study. Descriptive statistics like age, time from surgery and sport activity for the study participants are summarized in Table 2.

**Figure 3:**
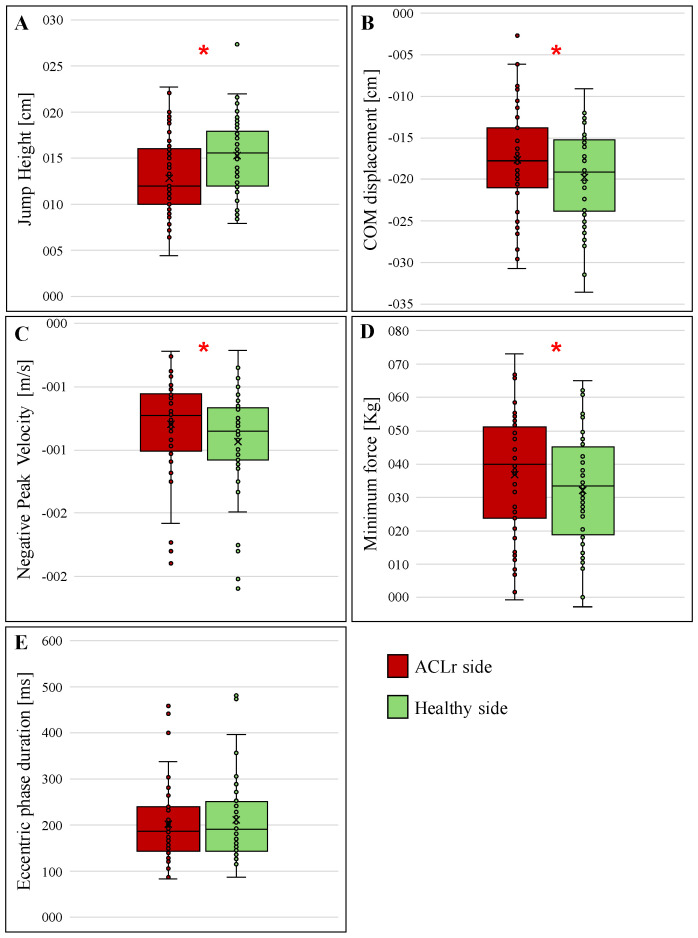
Comparison of countermovement jump (CMJ) metrics between the ACL-reconstructed limb (ACLr) and the contralateral healthy limb. (**A**) Jump height. (**B**) Center of mass (COM) displacement. (**C**) Negative peak velocity. (**D**) Minimum force. (**E**) Eccentric phase duration: no significant difference between limbs. Box plots represent group medians, interquartile ranges, and individual data points. * = significant differences *p* < 0.05.

The comparison of VJH between the healthy and ACLr (Figure 3) legs showed lower values in the ACLr leg compared to the healthy leg (12.9 ± 4.2 cm vs. 15.3 ± 4.0 cm; *p* < 0.001, respectively). Similarly, COM displacement was significantly lower in the ACLr leg compared to the healthy leg (−17.33 ± 4.46 cm vs. 21.40 ± 6.02 cm; *p* = 0.014) (Figure 3B). Negative peak velocity also differed significantly, with less negative values in the ACLr leg compared to the healthy leg (−0.80 ± 0.40 m/s vs. −0.94 ± 0.40 m/s; *p* < 0.001) (Figure 3C). Minimum force values were significantly higher in the ACLr leg than in the healthy leg (36.75 ± 17.88 N vs. 32.05 ± 17.25 N; *p* < 0.01) (Figure 3D). Finally, no significant differences were observed in eccentric phase duration between the ACLr and healthy leg (202.79 ± 83.76 ms vs. 212.33 ± 88.11 ms; *p* = 0.290) (Table 3; Figure 3E).

There were no significant differences in peak negative velocity across the groups defined by graft type (*p* = 0.943), sport (*p* = 0.647), TAS level (*p* = 0.238), and gender (*p* = 0.125). No significant differences were seen among these groups also for minimal force, jump height, COM displacement and eccentric phase duration.

## 4. Discussion

The objective of this study was to determine whether differences exist in the unweighting phase of the SLCMJ between the healthy and ACLr legs of patients after ACLr. Our findings revealed substantial differences in key metrics, specifically peak negative velocity, minimum force, and COM displacement, while no significant difference was observed in eccentric phase duration.

These findings are in line with previous research demonstrating that patients post-ACL reconstruction exhibit persistent asymmetries in vertical jump mechanics well beyond six months post-surgery. For example, Read et al. [25] reported that professional soccer players showed significant limb asymmetries in ground reaction force production and rate of force development up to 9 months post-ACLr, particularly during the eccentric loading phase. These asymmetries were associated with delayed return to performance and increased re-injury risk [25].

Unlike most studies that focus primarily on concentric outcomes (e.g., jump height, peak power), our study isolated specific eccentric-phase impairments, including reduced COM displacement and peak negative velocity in the ACL-reconstructed limb. These metrics suggest a compromised ability to absorb and store energy during the descent phase, which may reflect altered neuromuscular control or protective compensation strategies. Similar reductions in eccentric control and braking capability were previously linked to deficient stretch-shortening cycle mechanics in elite athletes [24].

The observed lower peak negative velocity in the ACLr leg compared to the healthy leg suggests an impairment in the ability to generate sufficient downward movement speed. This may be attributed to neuromuscular deficits, such as persistent quadriceps inhibition or altered motor control strategies, which limit the limb’s capacity to eccentrically control and store elastic energy efficiently [29]. This finding aligns with previous research highlighting deficits in eccentric strength and neuromuscular control after ACLr [20,23]. Reduced downward velocity may indicate a compensatory strategy to protect the ACLr limb by limiting force absorption demands during the deceleration phase of the SLCMJ. Similarly, the higher minimum force recorded in the ACLr leg suggests a reduced ability to fully unweight during the early descent phase. This could reflect an increased co-contraction of agonist and antagonist muscle groups, potentially driven by a fear-avoidance response or residual joint instability. The inability to achieve lower force values may also hinder the development of optimal stretch-shortening cycle mechanics, potentially impacting jump performance and overall neuromuscular efficiency [7,30].

The smaller COM displacement observed in the ACLr leg further supports the notion of a shallower movement pattern during the SLCMJ. This reduced depth may result from a lack of confidence in the ACLr leg, a protective mechanism to avoid excessive loading, or lingering biomechanical compensations. Shallow movement patterns have been associated with reduced capacity to utilize elastic energy storage, which can negatively impact overall jump performance and increase reliance on the healthy leg [18,31]. Although the absolute difference in COM displacement between limbs (~2.1 cm) may appear small, it is consistent with values previously reported in the literature and should not be underestimated in terms of clinical impact. For instance, Maestroni et al. [21] observed a ~2 cm reduction in peak COM displacement in the ACLr limb compared to the uninvolved limb during a single-leg vertical drop jump in professional soccer players approximately 8–9 months post-surgery (−0.18 ± 0.03 m vs. −0.20 ± 0.04 m), reflecting a “stiffer” knee movement strategy, which is also associated with an increased risk of re-injury [21]. In our sample, the reduced vertical excursion of the COM was accompanied by consistent alterations in other eccentric phase parameters, such as the negative peak velocity and minimum vertical ground reaction force, suggesting a globally modified motor control strategy in the ACLr limb. These asymmetries may have functional implications in terms of energy absorption, stretch-shortening cycle efficiency, and overall movement economy, especially in high-demand athletic tasks.

Interestingly, no significant differences were found in eccentric phase duration between the healthy and ACLr legs. This finding suggests that, while the ACLr leg may exhibit altered kinematic and kinetic strategies, the overall time taken to descend is not markedly different. This could imply that the patients have adapted their movement patterns to achieve similar temporal characteristics while compensating through alternative neuromuscular strategies.

From a clinical perspective, these findings are consistent with recent literature emphasizing the importance of limb symmetry as a criterion for safe return to sport after ACLr. A Limb Symmetry Index (LSI) of at least 90%, representing a difference between the operated and healthy limb of less than 10%, is commonly used to define sufficient functional recovery [32]. However, Legnani et al. [32] reported that only 21% of patients reached this level of symmetry at 6 months after ACLr, with the percentage increasing to 67% at 12 months (mean LSI 93%). This suggests that during the 6–9-month postoperative phase, a substantial proportion of patients still exhibit clinically meaningful asymmetries between limbs.

In our study, the observed difference of approximately 2.4 cm in VJH between the healthy and ACLr legs corresponds to a deficit of 10–15%, depending on overall jump performance. This exceeds the minimum clinically important difference (MCID) for SLCMJ height, which a previous study estimated to be around 2 cm [33]. Thus, the difference we observed is not only statistically significant but also clinically meaningful. Overall, our results underscore the persistence of functional asymmetries during the critical return-to-sport window and highlight the need to address these deficits to minimize re-injury risk and optimize outcomes.

These findings have important implications for rehabilitation practitioners aiming to optimize return-to-sport protocols after ACLr. The presence of altered unweighting strategies highlights the need for targeted interventions focusing on eccentric strength development, neuromuscular rehabilitation, and psychological readiness to load the ACLr leg effectively. Future research should explore whether these observed deficits persist beyond the 9-month post-ACLr period and whether they are predictive of re-injury risk or suboptimal performance outcomes. Longitudinal studies could provide valuable insights into the progression of neuromuscular adaptations over time and their relationship with functional recovery.

### 4.1. Limitations

Several limitations should be considered when interpreting these results. First, the retrospective design limits the ability to draw causal inferences, thereby weakening the interpretation of the observed associations.

Second, the absence of a healthy control group may restrict the generalizability of our findings. While emerging evidence suggests that compensatory adaptations can occur bilaterally after ACL reconstruction, potentially affecting neuromuscular control and jump mechanics in the uninvolved limb as well [34], the contralateral limb is frequently used as a reference in ACL research and clinical practice (e.g., for symmetry indices) [25,32].

A further limitation lies in our sample size estimation approach. Given the lack of normative data for the specific SLCMJ metrics and the absence of pilot data, sample size estimation was performed using a Monte Carlo simulation based on internal clinical experience and unpublished observations and was not derived from formal pilot data. While this method provided a reasonable basis for estimating variability and feasibility, it may reduce the statistical robustness of our sample size justification.

Furthermore, the sample was heterogeneous, including both male and female patients with different graft types (hamstring tendon, patellar tendon–bone–tendon, and quadriceps tendon), participation in various sports, and a wide range of activity levels (Tegner score 5 to 10). The limited sample size precluded the use of stratification or multivariate analyses to control for this variability, which may have introduced confounding factors. Future prospective longitudinal studies are needed to determine whether asymmetries persist over time and whether they are predictive of return-to-sport success or second injury events; furthermore, studies with larger and more balanced cohorts are warranted to explore potential gender-specific differences.

### 4.2. Practical Implications

**Figure 4:**
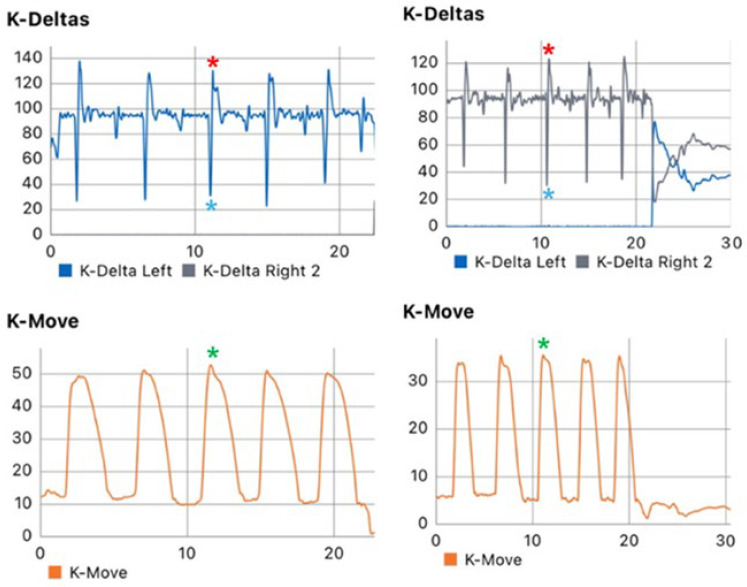
Difference in peak unweighting force, brake force and knee range of motion between right (ACLr) and left (healthy) leg post-ACLr. Red asterisks show peak brake force, blue asterisks peak unweight force and green asterisks peak knee range of motion. The left leg shows less peak unweight, brake force and knee range of motion. K-deltas indicate a force plate system, and K-move indicates a motion sensor device that could be applied on the knee for the assessment of flexion-extension range of motion.

Based on the findings of this study, we propose an evaluation and treatment model specifically targeting the descent phase of dynamic movements, such as the SLCMJ. For the assessment component, we recommend the use of force plates to analyze vertical ground reaction force profiles, enabling detailed evaluation of load absorption, energy storage, and braking capacity during the eccentric phase of a SLCMJ. For the treatment and monitoring phases, we suggest the combined use of force plates (for example, K-Dealtas, Kinvent, Montpellier, Fance) to compare load symmetry between the ACLr and unACLr limbs and motion sensors (for example, K-move, Kinvent, Montpellier, Fance) to assess joint-specific range of motion (Figure 4).

**Figure 5:**
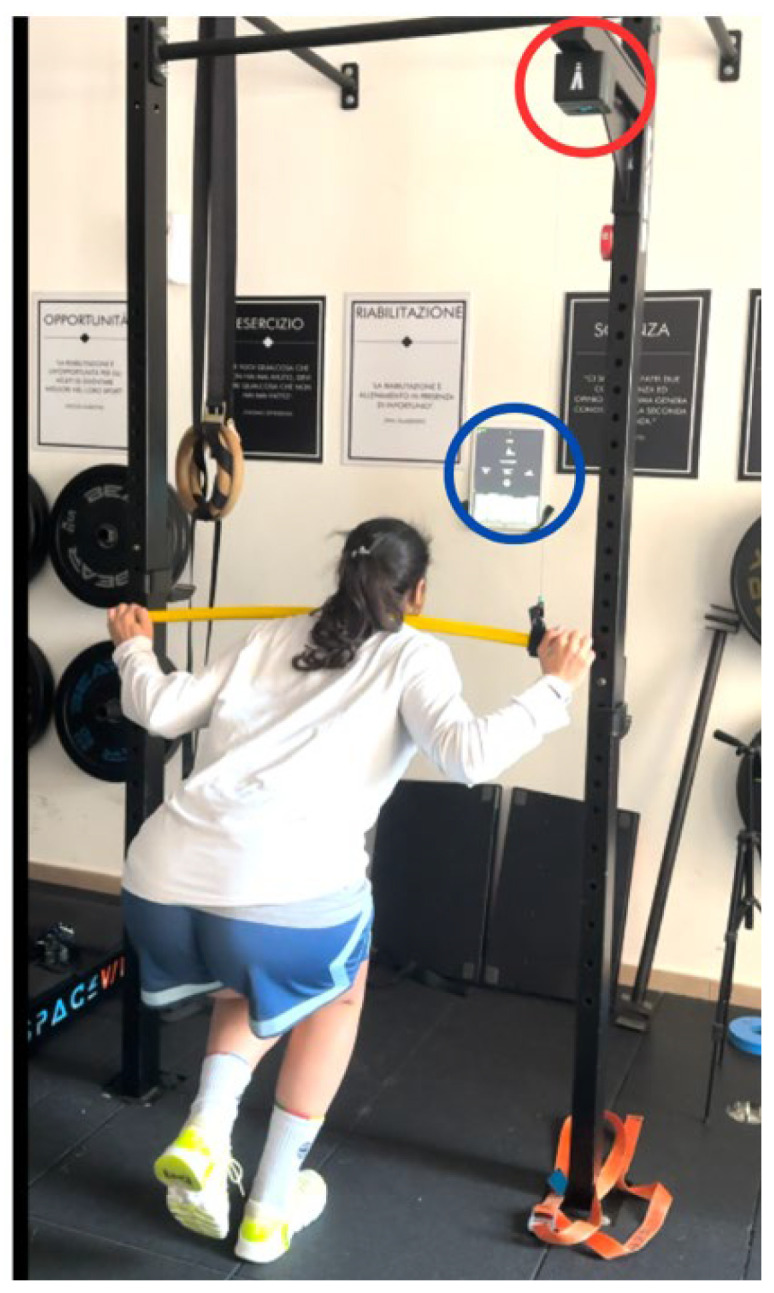
Drop single-leg squat with encoder. The red circle shows the encoder positioned at the top of the rack, and the blue circle indicates the peak negative velocity.

To measure negative velocity, we employ a linear position transducer oriented from top to bottom, rather than bottom-up, to more accurately capture downward movement velocity. An example of an exercise utilizing this setup is the drop single-leg squat, as depicted in Figure 5.

From a clinical standpoint, these findings support the inclusion of eccentric overload exercises and braking drills in ACL rehabilitation, with an emphasis on symmetry restoration not only during propulsion but also during the initial descent. Furthermore, force plate assessments focused on the unweighting phase may offer a sensitive method to detect residual deficits that traditional jump height comparisons could miss. Future work should examine whether persistent unweighting-phase asymmetries predict functional outcomes or re-injury risk beyond the 9-month period.

## 5. Conclusions

In conclusion, this study offers new information about the unweighting phase of the CMJ in patients after ACLr. Results showed that the ACLr and healthy limbs differ significantly in terms of peak negative velocity, minimum force, and COM displacement. These findings highlight the importance of assessing the early phases of the jump to better inform rehabilitation strategies and improve functional recovery after ACLr.

## Figures and Tables

**Table 1:** SLCMJ testing procedures.

Phase	Description
Generalized warm-up	10 min exercise bike
Specific warm-up	2 sets of 10 bilateral squats, lunges and push-ups (total sets and reps are 6 and 60, respectively)
Familiarization trials	2 SLCMJ trials with each leg
Indications	Arms on hips, leg in the front at 45° hip flexion, no swing accepted
Execution order	First, healthy leg
Cue	“Go down and up as fast as you can and jump as high as you can”
Number of trials	2
Value	Best of two
Rest between trials	30 s

**Table 2:** Descriptive statistics of the participants.

Characteristics	Total Sample (n = 53)
Age (Yr), mean ± SD	25.98±5.58
*Gender*	
Male, n (%)	41 (78.8)
Female, n (%)	11 (21.2)
Weight (Kg), mean ± SD	78.94 ± 8.85
Height (cm), mean ± SD	177.94 ± 7.77
*Graft type*	
R HT/G, n (%)	26 (50.0)
L HT/G, n (%)	20 (38.5)
R PTBT, n (%)	4 (7.7)
L PTBT	1 (1.9)
Quadriceps (QT), n (%)	1 (1.9)
Time from surgery (months), mean ± SD	7.73 ± 0.95
*Sport Activity*	
Soccer, n (%)	20 (38.5)
Basketball, n (%)	12 (23.1)
Volleyball, n (%)	7 (13.5)
Futsal, n (%)	3 (5.8)
CrossFit, n (%)	3 (5.8)
Surf, n (%)	2 (3.8)
Box, n (%)	2 (3.8)
Ski, n (%)	1 (1.9)
Touch rugby, n (%)	1 (1.9)
Surf, n (%)	1 (1.9)
*TAS level,* mean ± SD	7.46 ± 1.58

Yr: years, SD: standard deviation, n: number, %: percentual of total population, Kg: kilogram, cm: centimeters, R: right, L: left, HT/G: hamstrings tendon/gracilis tendon, PTBT: patellar tendon bone tendon, QT: quad tendon, TAS: Tegner Activity Scale.

**Table 3:** Jump metrics of the SLCMJ.

Jump Metrics	Total Sample (n = 53)		*p*-Value
	ACLr Side	Healthy Side	
Height (cm), n ± SD	12.90 ± 4.22	15.27 ± 4.03	<0.001 *
COM displacement (cm), n ± SD	−17.62 ± 6.25	−19.73 ± 5.34	0.014 *
Negative velocity peak (m/s), n ± SD	−0.80 ± 0.40	−0.94 ± 0.40	<0.001 **
Minimum force (Kg), n ± SD	36.75 ± 17.88	32.05 ± 17.25	<0.001 *
Eccentric phase duration (ms)	202.79 ± 83.76	212.33 ± 88.11	0.290 **

* Student’s *t*-test; ** non-parametric Wilcoxon test.

## Data Availability

The data supporting the findings of this study are available from the corresponding authors upon reasonable request.

## Abbreviations

The following abbreviations are used in this manuscript:

ACLr	anterior cruciate ligament reconstruction
ACL	anterior cruciate ligament
BW	body weight
CMJ	countermovement jump
COM	center of mass
MCID	minimum clinically important difference
SD	standard deviation
SLCMJ	single-leg countermovement jump
TAS	Tegner Activity Score
VJH	vertical jump height
